# Understanding the Inequality of Web Traffic and Engagement in Online Healthcare Communities

**DOI:** 10.3389/fpubh.2022.917522

**Published:** 2022-06-07

**Authors:** Yuan-Teng Hsu, Ran Duan, Ya-Ling Chiu, Jying-Nan Wang

**Affiliations:** ^1^Research Center of Finance, Shanghai Business School, Shanghai, China; ^2^School of Economics and Management, Chongqing University of Posts and Telecommunications, Chongqing, China; ^3^College of International Business, Zhejiang Yuexiu University, Zhejiang, China; ^4^Shaoxing Key Laboratory for Smart Society Monitoring, Prevention & Control, Shaoxing, China

**Keywords:** online healthcare community, Gini index, Matthew effect, reward mechanism, inequality

## Abstract

The online healthcare community (OHC) is a kind of doctor-patient communication platform, in which doctors can share medical knowledge and provide various kinds of counsel for patients. However, if the OHC's web traffic is concentrated on a small number of doctors, or if only a few doctors are actively involved in the OHC's activities, this will not be conducive to the optimal development of the OHC. This study explores this issue of inequality and makes three main innovations. First, based on data on web traffic and engagement extracted from 139,037 doctors' web pages in one popular OHC, we point out how serious the inequality phenomenon is. Second, we confirm that the Matthew effect indeed exists in this context and leads to greater inequality. Third, we demonstrate that the inequality of psychological or material rewards causes the inequality of web traffic or engagement to become worse; hence, an appropriate reward mechanism should be designed to mitigate the Matthew effect rather than enhance it. Finally, we discuss the managerial implications of these results, as well as avenues for future studies.

## 1. Introduction

With the popularity of online communities in various fields (1–4), individuals have become more likely to obtain information they require on the Internet and participate in the generation of content. The online healthcare community (OHC) is a special case in point. In general, OHCs allow members of the public to share their opinions or experiences about the medical treatment process, for example, by making comments about doctors, which indeed helps with the transmission of information about the quality of services (5–8). Unlike with other types of online communities, since most of the content in OHCs directly affects an individual's health, people generally pay careful attention to the reliability of the content on OHC sites. The easiest way to eliminate concern about the reliability of this content is to make sure the information provider is a doctor. Therefore, whether doctors actively participate in OHC activities such as sharing medical knowledge, communicating with patients, or even providing counseling and whether such kinds of engagement can attract more patients has a key effect in influencing the development of OHCs (9, 10). With the purpose of making possible greater understanding of the role of doctors in OHCs, this study explores the distribution of two important features of such doctors: the web traffic related to the doctor's page and the level of the doctor's online engagement.

It is worthwhile to further comment on the importance of the two mentioned features for online communities. First, from the economic viewpoint, web traffic can be used to assess a firm's value (11–15). Specifically, if more people visit a firm's website, that indicates that more people have received information related to the firm's products or services. In other words, greater traffic enhances the odds of conversion from interest to consumer purchases, which leads to higher firm sales (2, 16). From the marketing point of view, higher web traffic usually increases brand recognition, which will attract more new users and make the switching costs for registered users higher (17). Moreover, since there is a significant relationship between web traffic and firm value, mediated by word-of-mouth (18), exploring web traffic is helpful in the effort to understand the online marketing effort. The other feature is the level of online engagement, which can be measured by whether the individual's participation in the online community is active. A stream of studies have investigated online engagement behavior in several kinds of online communities such as Wikipedia (19, 20), open source software communities (21, 22), and social Q&A sites (23–25). The active participation of members in the community is a key factor for the sustainable development of an online community (26, 27). Because this study is aimed at the OHC, we focus on the web traffic and online engagement associated with doctors, rather than with general community members.

Given the importance of web traffic and engagement, the main innovations of this study can be seen in relation to the following three points. First, we analyzed a data set of 139,037 doctors from the Haodf website, which is the most popular OHC in China, to discover how serious inequality in the OHC is, in terms of the doctors' web traffic and engagement. The inequality phenomenon can be illustrated by the Pareto principle^1^, also known as the 80/20 rule, which claims that roughly 80% of the effects come from 20% of the causes, and has been applied in various fields, e.g., economics, business, biology, and criminology (29). This study verifies whether or not the Pareto principle applies in the context of the OHC. Severely inequitable distribution in the doctors' web traffic and engagement might bring with it some problems in the development of OHCs. For example, if a small number of doctors account for the majority of web traffic, it would imply most doctors have a small amount of traffic, and this might cause them to lose interest in OHC activities. From the engagement perspective, if only a few doctors are actively involved in the activity, the diversity of information that the community can provide will be limited.

Second, in order to understand the cause of inequitable distribution of OHC web traffic and engagement, we use the Matthew effect to comprehensively explain these observed distributional characteristics. Inspired by the biblical Gospel of Matthew, (30) first used this term to describe the phenomenon that famous scientists often obtain more credit than lesser-known ones for similar work. Its secular expression is the rich tend to get richer and the poor tend to get poorer. Following this concept, we examine whether a doctor with more web traffic would keep this advantage and continue to receive more web traffic in the future. Similarly, when a doctor has had more engagement in an OHC, we also expect him to continue engaging more than others who have had less previous engagement. Based on our empirical findings, we conclude that the Matthew effect is significant in the OHC and leads to inequitable distributions of doctors' web traffic and engagement. However, OHC managers, even if they know that the Matthew effect causes inequality, have no explicit means to ameliorate this problem. Therefore, the third innovation of this study is to make suggestions regarding what the managers might do in regard to the reward mechanism, including psychological and material rewards (31–33). We assigned doctors to different groups according to their expertise with various diseases. Then we designed regression models to study this problem. Having considering several reasonable control variables, we conclude that the more inequitable the rewards in a certain group are, the more inequitable the web traffic and engagement will be.

In the remainder of this paper, we first review the existing literature related to this study and develop the research hypotheses. Section 3 describes the data collection process and the measurement of the variables. In Section 4, we conduct data analysis to verify whether inequality of increased web traffic or increased engagement exists in the OHC and then explore whether such inequality is caused by the Matthew effect. Moreover, regression models are used to examine the effect of the reward mechanism. Finally, we discuss the management implications, research limitations, and future research directions.

## 2. Literature Reviews and Hypotheses

### 2.1. Online Healthcare Community Development Status

Participants in OHCs can be divided into three main groups: patients, doctors, and community managers. From a perspective on patients, some studies have confirmed that OHCs can improve individuals' lives, taking advantage of the Internet (34–37). A stream of research focuses on online reviews of doctors, which can help patients obtain information about their doctors (5, 38) and on how differences in medical specialty areas affect these online reviews (7, 39). From a perspective on doctors, since the sustainability of the OHC relies on the participation of doctors, (10) find that both psychological and material rewards can make doctors more willing to engage in OHC activities. Moreover, Guo et al. (9) use social exchange theory (40) to argue that doctor participation in OHCs is a social exchange behavior and to examine the determinants of the social and economic returns of doctors. However, as far as we know, there is no research which has explored the overall situation of the OHC from a perspective on community managers or providers. This study attempts to fill this gap.

Due to the importance of doctors' web traffic and engagement as mentioned in Section 1, OHC managers should be aware of the distributed inequality for both factors, which is consistent with the Pareto principle and can be expressed mathematically as a power law. Indeed, this pattern of inequality on the Internet has been verified in many studies. The empirical outcomes show that millions of users crowd to certain specific websites and pay little attention to millions of other websites (41, 42). In addition, looking at online community engagement, many studies have pointed out that a small share of participators makes up a large share of the engagement in online communities such as Open Source Software and Wikipedia (43–45). In particular, Sauermann and Franzoni (46) analyze data from seven projects in a crowd science community and conclude that top 10% of the contributors made between 71 and 88% of all contributions. Similar traffic inequality exists for e-commerce and video sites. In short, it is not surprising that a similar phenomenon should appear in OHCs. Our empirical results not only verify the reality of these factors but also reveal how serious these imbalances are in OHCs, in terms of doctors' web traffic and engagement.

### 2.2. The Matthew Effect

The Matthew effect relates to the common recognition that those who already have an advantaged status are often in a place in which they can gain more, while those who do not have an advantaged status often have difficulty getting more. In fact, this concept has been applied widely for a long time and is closely related to several other concepts in the natural sciences. For example, the Yule process indicates that the distribution of each species typically exhibits power-law behavior in the evolution of biological populations (47, 48). In other words, an initially small number of advantages may snowball and become greatly significant over time. The Matthew effect is also often used to explain the phenomenon of inequality or unevenness in many aspects of our social life. McMahon (49) points that income distribution in the United States shows a strong Matthew effect in line with an increasing concentration of income and wealth in the top 1% over the years, but the federal tax system has not been able to solve this problem. In education, the Matthew effect can be used to illustrate the difference in learning outcomes; for example, early success in acquiring reading skills often leads to subsequent reading success as children grow, and failure to learn early may lead to a reading ability gap that increases over time (50, 51). In social commerce, using a dataset of 2,187 reviews from six best-selling products on amazon.com, Wan (52) finds that early-posted reviews are more likely to obtain a disproportionately higher percentage of votes due to the Matthew effect.

Since the origin of the Matthew effect is generally very complex, there is no simple way to explain it entirely (53). We propose some intuitive arguments separately to illustrate why the Matthew effect exists in the doctor's web traffic and engagement. First, for web traffic, a doctor who acquires more traffic than another will increase her or his popularity at a higher rate, and thus an initial difference in the traffic between two doctors will increase further along with the development of the OHC. This argument is also close to that involved in the well-known first mover advantage in marketing (54). In other words, when doctors join the OHC earlier or have a longer tenure with the OHC, it is easy for them to attain an advantageous status, in which they can attract more patient attention and generate more web traffic. Indeed, no matter how long ago a doctor has joined the OHC, as long as she or he has already received more cumulative web traffic by any means, she or he will achieve more traffic in the next days. This phenomenon can be explained in terms of signal theory. Due to the information asymmetry in OHC, patients can usually only rely on word-of-mouth to find high-quality physicians. When data on physician web traffic can be observed by patients, high traffic is more likely to be perceived by patients as positive word-of-mouth. This will make it easier for physicians who have accumulated more web traffic to receive more traffic. Based on previous discussion and analysis, we propose the following two hypotheses:

**Hypothesis 1a:** *The tenure of a doctor will be positively associated with increased web traffic in the subsequent period*.
**Hypothesis 1b:** *A doctor's cumulative web traffic up to and including the prior period will be positively associated with increased web traffic in the subsequent period*.

We futher explore whether, similar to web traffic, the doctor's tenure and cumulative engagement are related to her or his increased engagement in the subsequent period. A member's tenure is commonly regarded as one of the potential determinants of engagement in virtual communities (24, 55). Since members' tenures can be seen as cumulative investments in the community, members may be expected to continue to participate in community activities to maintain the status quo (56). Following this rationale, tenure is expected to have a positive effect on engagement. However, the empirical results are mixed; in many cases, tenure does not significantly influence engagement (57, 58). One of the possible reasons is that members' interest may fade as their initial enthusiasm subsides; as a result, they may become lurkers (59, 60). Since the former of the two descriptions above is more consistent with the concept of the Matthew effect, we adopt it in our effort to explain the impact of tenure on engagement and propose the following hypothesis:

**Hypothesis 2a:** *The tenure of a doctor will be positively associated with increased engagement in the subsequent period*.

Researchers also pay attention to the influence of habits in community engagement (61–64). Briefly, the online environment facilitates the formation of user habits as shown in the high correlation between past use and future use (62). That is to say, when someone continues to participate in community activities, she or he will gradually become habitual in participating in these activities as part of daily life without having to consciously plan to do so (24). In our context, if online engagement, such as sharing medical knowledge or responding to patients' questions, becomes a doctor's habitual behavior, she or he will be more likely continue to engage in the future. Moreover, greater cumulative engagement implies higher cumulative investments in OHCs, which may also make doctors wish to maintain their online status. Therefore, we propose the following hypothesis:

**Hypothesis 2b:** *A doctor's cumulative engagement up to and including the prior period will be positively associated with increased engagement in the subsequent period*.

### 2.3. Reward Mechanism

As indicated in the previous discussion, we have concluded that the Matthew effect may induce the phenomenon of inherent inequality in many social and business activities and that such inequality directly reveals the problem of inequitable resource allocation. In this context, OHC managers should not ignore the question of how to mitigate the impact of the Matthew effect on doctors' web traffic and engagement. Although the Matthew effect involves problems that are difficult to solve due to its mixed causes with relation to human nature, a stream of studies have confirmed that individuals' behavior can be shaped and modified through the design of a website (33, 58). Therefore, this study investigates whether this kind of inequality in OHCs can be ameliorated through developing appropriate reward mechanisms including psychological and material rewards (9, 10). Specifically, OHC managers can offer a platform for patients to express their gratitude to doctors for sharing knowledge, answering questions, or providing counseling.

Taking the Haodf website as an example, doctors may receive thank you letters that can enhance their online reputation as they are widely recognized by patients and thus make doctors feel that they are valued by public (65). Hence, the thank you letter is regarded as a kind of psychological reward. In addition, patients might present doctors with token gifts, which can be converted into cash equivalents and deposited into the doctor's personal research fund. Thus, the token gift is regarded as a kind of material reward. Two important points should be noted. First, the number of rewards that doctors have received in the past is available to the public, so this number essentially represents the reputations of doctors and thus significant effects on patients' trust levels (66, 67). It stands to reason that doctors with better reputations will usually be able to attract more patients in an OHC. Second, a reward mechanism usually plays a vital role in influencing community engagement (68, 69). In particular, Wang et al. (10) show that both psychological and material rewards can lead to a significant increase in a doctor's online engagement. Moreover, material reward has a larger influence than psychological reward. Based on previous literature, we believe that more rewards can bring more web traffic and motivate doctors to participate more actively in OHCs. In other words, an appropriate distribution of rewards will help to improve the aforementioned inequalities. Accordingly, we propose the following two hypotheses:

**Hypothesis 3a:** *When the number of psychological or material rewards received by a group of doctors is more inequitable, the distribution of their increased web traffic will be more inequitable*.
**Hypothesis 3b:** *When the number of psychological or material rewards received by a group of doctors is more inequitable, the distribution of their increased engagement will be more inequitable*.

## 3. Data and Variables Measurement

### 3.1. Data Collection

The data were collected from the Haodf website (hao means “good,” and dai fu means “doctor” in Chinese), which is one of the most popular OHCs in China. Due to its representation, it has been used to explore various OHC topics (7, 9, 10). From July 26, 2017, to July 28, 2017, we used web crawler technology to survey more than 423,000 doctors' profiles on the Haodf website (www.haodf.com). However, the fact that a doctor's profile appears on the Haodf website does not mean she or he really engages in this OHC: doctors' profiles might be added by Haodf employees at whim. Only when doctors complete their identity authentication and activate their own website by themselves can they actually begin to share knowledge or provide other consulting services. Owing to the needs of this research, the doctors included in our sample must have activated their personal web pages, and their web traffic in the subsequent month must be greater than zero. Additionally, in order to conduct a longitudinal study involving three-wave data samples, we collected data every other month starting from July 26, 2017, and we collected the data a total of three times. If the doctor's data for these 3 months were not complete, we also removed them from the sample. Finally, we further deleted a small amount of data containing unreasonable values; for example, two doctors had negative web traffic, an impossible value. Following the completion of the filtering procedure described above, a sample of 139,037 doctors was considered for our analysis.

### 3.2. Variable Measurement

All information required for measuring variables is available on each doctor's web page. We detail the definitions of these variables in this section. First, each doctor's web traffic was measured via the number of visits that had accumulated since her or his web page was activated. Whenever one patient entered one doctor's web page, regardless of whether the purpose was to obtain the doctor's personal information or medical knowledge, the number of visits would increase by one. Therefore, at time *t*, the cumulative web traffic (*WT*_*t*_) is defined as the cumulative number of visits to the doctor's web page. Moreover, we also define the increased web traffic (Δ*WT*_*t*_) as an increase in the number of visits to the doctor's web page from *t*−1 to *t*, i.e., Δ*WT*_*t*_ = *WT*_*t*_−*WT*_*t*−1_. The doctor's engagement was measured through the contribution scores^2^ reported on the Haodf website. The contribution score is composed of three parts. First, when a doctor updates her or his personal information, such as outpatient information, her or his contribution score will increase as a result of an administrator's review. Second, the Haodf website hopes doctors will share medical knowledge with patients more frequently. Therefore, posting article will also increase the doctor's contribution score. Third, when a doctor answers patients' questions online, the number of responses is included in the calculation of the contribution score. Therefore, for each doctor, we can define the cumulative engagement (*EG*_*t*_) as the doctor's contribution score on the Haodf website at time *t* and the increased engagement (Δ*EG*_*t*_) as the difference in the doctor's contribution scores on the Haodf website between *t*−1 and *t*, i.e., Δ*EG*_*t*_ = *EG*_*t*_−*EG*_*t*−1_.

There are four other variables that will be subject to further analysis. First, tenure with the Haodf (TENURE_*t*_) for a doctor at time *t* is calculated by the data download date minus the date on which this doctor activated pages on the website. A larger value for this variable means that a doctor has had web pages activated for a longer time. The doctor's having had web pages activated earlier implies she or he is more likely to have obtained the first mover advantage (54). Second, we measure the doctor's online reputation via the mean of overall ratings on the Haodf website, which is denoted as RATING_*t*_. The rating of the patient review can usually represent the quality of the doctor reviewed (70–72), and this helps the patient to choose an appropriate doctor more easily. The other two variables are related to the reward mechanism. Patients can express gratitude to doctors by writing thank you letters. Since doctors might be encouraged by these letters, the number of thank you letters received is used as a proxy for psychological reward. Besides thank you letters, patients can also deliver gratitude to doctors by purchasing virtual gifts such as virtual flowers or plaques on the Haodf website. Since these gifts can be converted into cash equivalents and are deposited into the doctor's personal research fund, this study regards the number of token gifts as a proxy for material reward. Formally, we define Δ*PR*_*t*_ and Δ*MR*_*t*_ as the numbers of thank you letters and token gifts, respectively, the doctor has received from time *t*−1 to *t*. The definitions and measurements of all variables are reported in Table 1. More detailed descriptions of group-level variables will be given in Section 4.4.

**Table 1:** Variable definitions.

**Variable**	**Symbol**	**Measurement**
**Individual-level variables**		
Cumulative web traffic	*WT*_*t*_	Cumulative web traffic is measured by the cumulative number of visits to the doctor's personal web page at time *t*.
Increased web traffic	Δ*WT*_*t*_	Increased web traffic is measured by the increased number of visits to the doctor's personal web page from time *t*−1 to *t*.
Cumulative engagement	*EG*_*t*_	Cumulative engagement is measured by the the doctor's contribution score on the Haodf website at time *t*.
Increased engagement	Δ*EG*_*t*_	Increased engagement is measured by the difference of doctor's contribution scores on the Haodf website between time *t*−1 and *t*.
Tenure with the Haodf	TENURE_*t*_	The doctor's tenure with the Haodf website (days) is calculated by data download date minus this doctor's activating date on the website at time *t*.
**Group-level variables** ^a^		
Gini index of increased web traffic	Gini($\Delta WT_{t}^{*}$)^b^	This represents the Gini index for the distribution of the adjusted increased web traffic from time *t*−1 to *t*.
Gini index of cumulative web traffic	Gini($WT_{t}^{*}$)^b^	This represents the Gini index for the distribution of the adjusted cumulative web traffic at time *t*.
Gini index of Increased engagement	Gini($\Delta EG_{t}^{*}$)^b^	This represents the Gini index for the distribution of the adjusted increased engagement from time *t*−1 to *t*.
Gini index of cumulative engagement	Gini($EG_{t}^{*}$)^b^	This represents the Gini index for the distribution of the adjusted cumulative engagement at time *t*.
Group size	SIZE_*t*_	The group size is the number of doctors belonging to the group at time *t*.
Average ratings	Avg(RATING_*t*_)	The average value of doctors' review ratings in one specific group at time *t*.
Average tenure with haodf	Avg(TENURE_*t*_)	The average length of doctors' tenures in one specific group at time *t*.
Psychological reward Gini index	Gini(Δ*PR*_*t*_)	The Gini index for the distribution of Δ*PR*_*t*_ is calculated by how many thank you letters the doctor received from time *t*−1 to *t*.
Material reward Gini index	Gini(Δ*MR*_*t*_)	The Gini index for the distribution of Δ*MR*_*t*_ is calculated by how many token gifts the doctor received from time *t*−1 to *t*.

*All variables are available from the Haodf website and have been de-identified to preserve personal privacy*.

a *When a doctor has received at least one vote for a specific disease j on the Haodf website, we assign this doctor into the group j. In this way, each doctor might belong to zero or more than one disease type groups. Each group-level variable is measured by the characteristics of the doctors within one corresponding group*.

b According to the number of votes given by patients to the doctor for different disease types, we calculate the adjusted cumulative (or increased) web traffic (or engagement) by the vote weights. The superscript ^*^ is used to indicate that a variable has been adjusted by the vote weights.

## 4. Data Analysis

### 4.1. Descriptive Statistics

**Figure 1:**
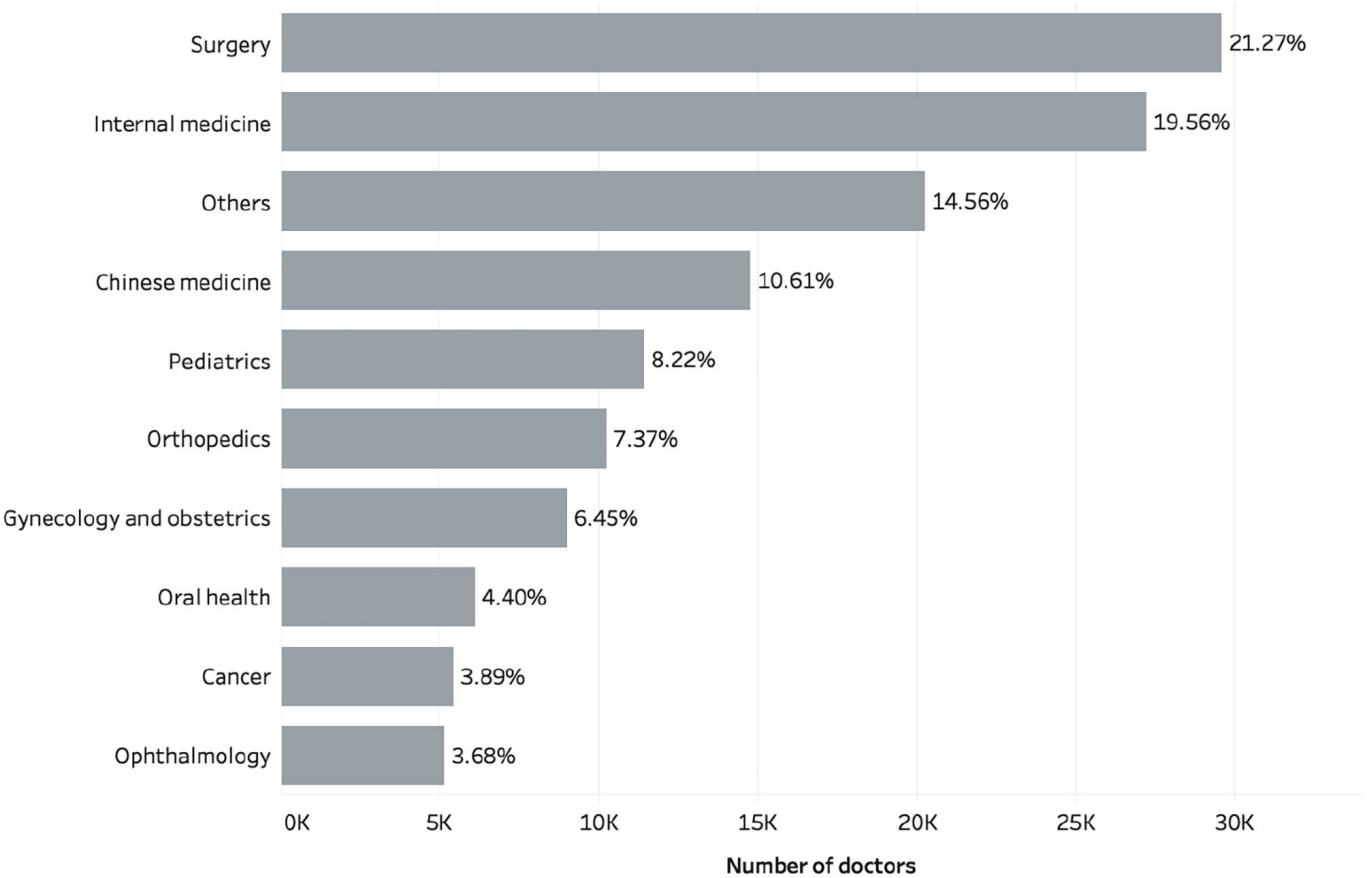
Number of doctors in 10 major specialty areas.

**Figure 2:**
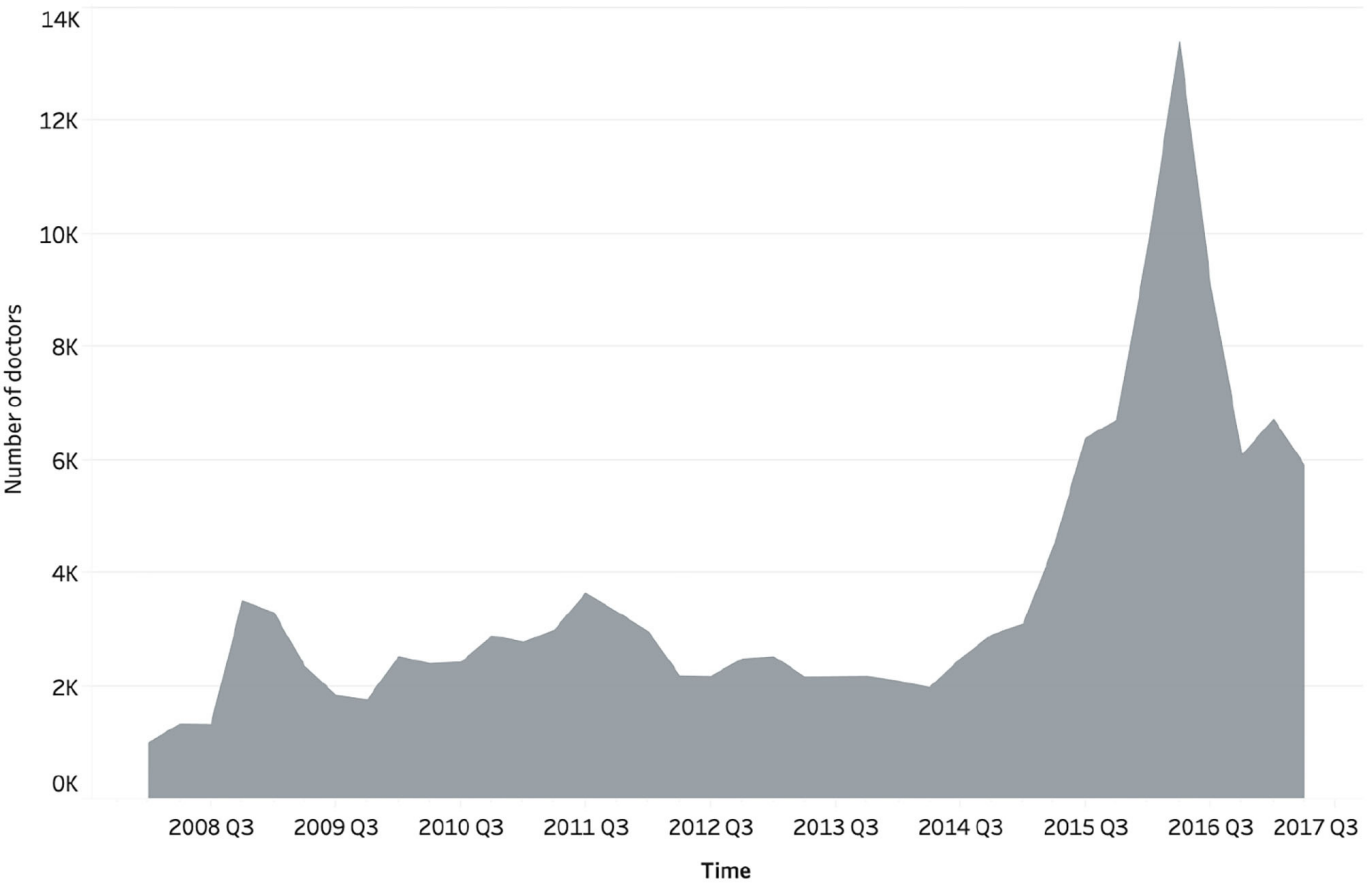
Number of doctors activating the web pages in different quarters.

It is worthwhile to investigate certain sample characteristics which show that the effects of sample selection bias are limited in this study. Our sample consists of 139,037 doctors from 6,549 varied hospitals, 10 different specialty areas, and 31 provinces or municipalities in China. Figure 1 shows the numbers of doctors in the 10 major specialty areas, of which surgery, internal medicine, Chinese medicine, and pediatrics are the largest four groups, accounting for 21.27, 19.56, 10.61, and 8.22% of all doctors, respectively. In addition, to verify the impact of the doctor's tenure on website traffic and engagement, we also checked to see if the doctor had activated her or his web page on a different date. Based on the whole sample, Figure 2 presents the number of doctors activating web pages in different quarters. In each quarter of 2008–2014, the number of doctors who activated personal web pages was relatively stable, generally increasing by 990–3,632 doctors per season, but there was a relatively significant increase in the number of people from 2015 to 2017, especially for the second quarter of 2016, in which 13,390 doctors activated web pages. The reason for the difference in the number of doctors in each season is difficult to discern through our sample. For example, a small observed number of doctors in the early period may be due to some doctors having already withdrawn from the Haodf website, or a larger number of doctors in the later period may have been caused by the expansion of the website. Even so, the above characteristics still indicate our sample is not only confined in its relevance to a specific group but likely reflects the current state of OHCs in China.

Since we are conducting a longitudinal study, data collected from Aug 26, 2017 to Aug 28, 2017 are taken as the portion of the sample from time *t*−1. After 1 month, from September 25, 2017 to September 27, 2017, we collected data again and used them as the portion of the sample from time *t*. Table 2 reports the descriptive statistics for web traffic and engagement. The means of the cumulative and increased web traffic are significantly larger than the medians, implying that their distributions show positive skewness. The cumulative and increased engagements have characteristics similar those of web traffic, except for up to 66.7% of doctors, who showed zero increased value. Moreover, all of the maximums are much larger than the means. These results preliminarily indicate that the distributions of the doctor's increased web traffic and engagement are extremely inequitable. To elaborate on how serious this phenomenon is, we provide further evidence to verify our arguments.

**Table 2:** Descriptive statistics for web traffic and engagement.

	**Mean**	**Median**	**Std.dev^*a*^**	**Max**	**min**
Cumulative web traffic at *t*−1	239,568	17,551	1,410,284	122,773,600	93
Cumulative web traffic *t*	246,548	18,336	1,436,946	124,880,400	165
Increased web traffic	6,980	610	31,433	2,106,824	24
Cumulative engagement at *t*−1	3,469	125	16,139	1,316,059	0
Cumulative engagement at *t*	3,547	135	16,356	1,320,579	0
Increased engagement	79	0	397	25,875	0

*Data collected from Aug 26, 2017, to Aug 28, 2017 are regarded as the sample for time t−1. After 1 month, from September 25, 2017, to September 27, 2017, we recollected the data and represented them as the sample for time t*.

a *Std.dev means the standard deviation*.

### 4.2. Evidence of Inequitable Distribution

**Figure 3:**
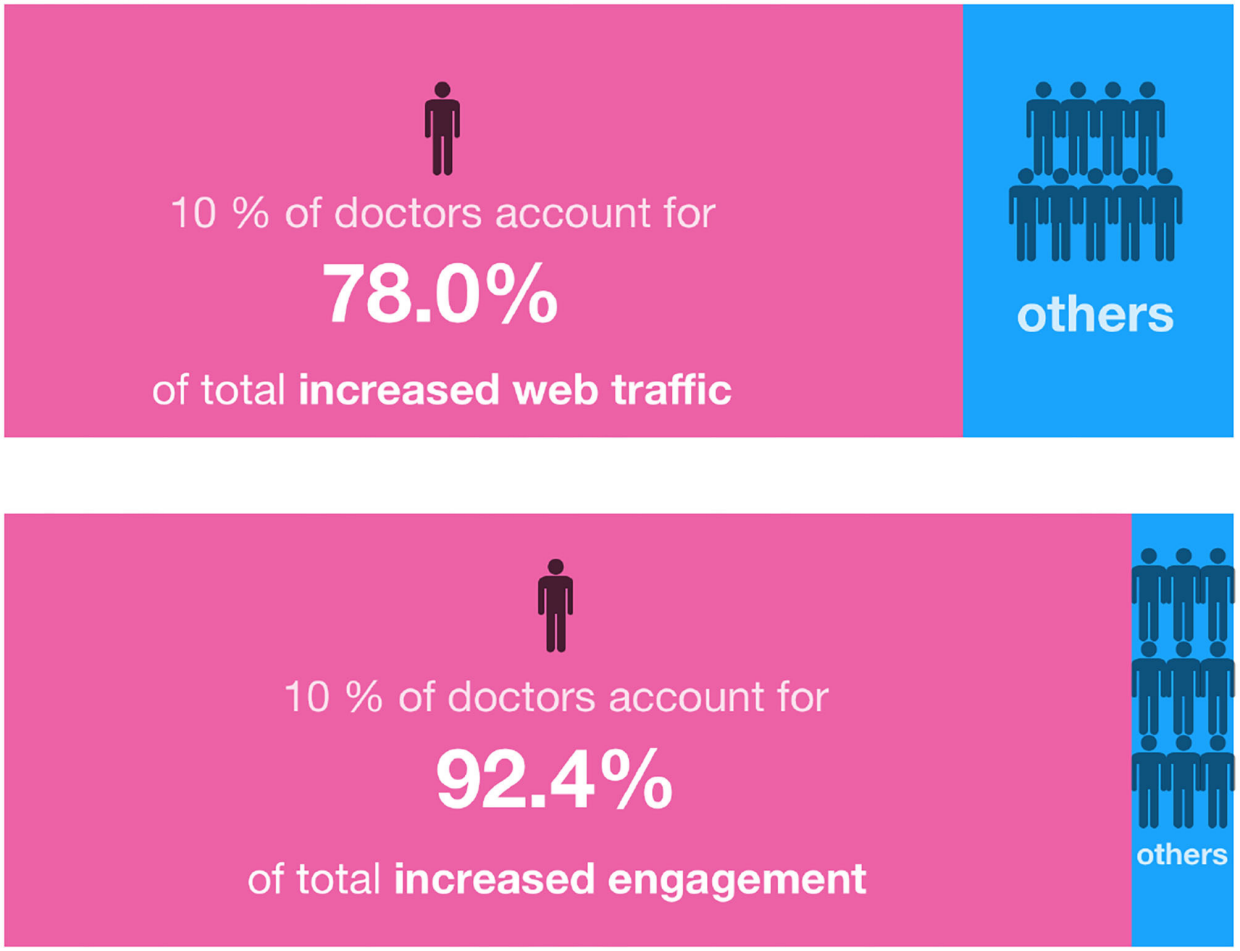
The inequality of increased web traffic and increased engagement.

Because we are primarily concerned about the sustainable development of OHCs, this study focuses on the inequality of increased web traffic and engagement, rather than the inequality of cumulative values. First, we point out the degree of inequality in Figure 3. The data indicate that the top 10% of doctors, ranked by web traffic or increased engagement, account for 78.0% of the total increased web traffic and 92.4% of the total increased engagement. In short, a small number of doctors attract a large number of patients' attention and thus create most of the web traffic. To understand the characteristics of high-traffic doctors, we further analyzed these top 10% doctors and found that the total number of doctors from tertiary hospitals accounted for 84% of the total increased web traffic and 85.51% of the total increased engagement. Among them, the proportion of chief physicians is 41.25% of the total increased web traffic and 31.01% of the total increased engagement, and the proportion from large cities (Beijing, Shanghai, Guangzhou, Shenzhen) is 36.60% of the total increased web traffic and 33.02% of the total increased engagement. Doctors in tertiary hospitals, chief physicians or from large cities will have a more significant effect. In addition, the development of OHCs depends on the engagement of doctors, but only a relatively few doctors actively engage in community activities. Accordingly, from the perspectives of web traffic and engagement, there are a few vital doctors alongside others who are relatively insignificant in this OHC. Even if we consider only the top 1% of doctors in terms of increased web traffic and engagement, they still account for 32.7 and 38.2% of the total values, respectively.

**Figure 4:**
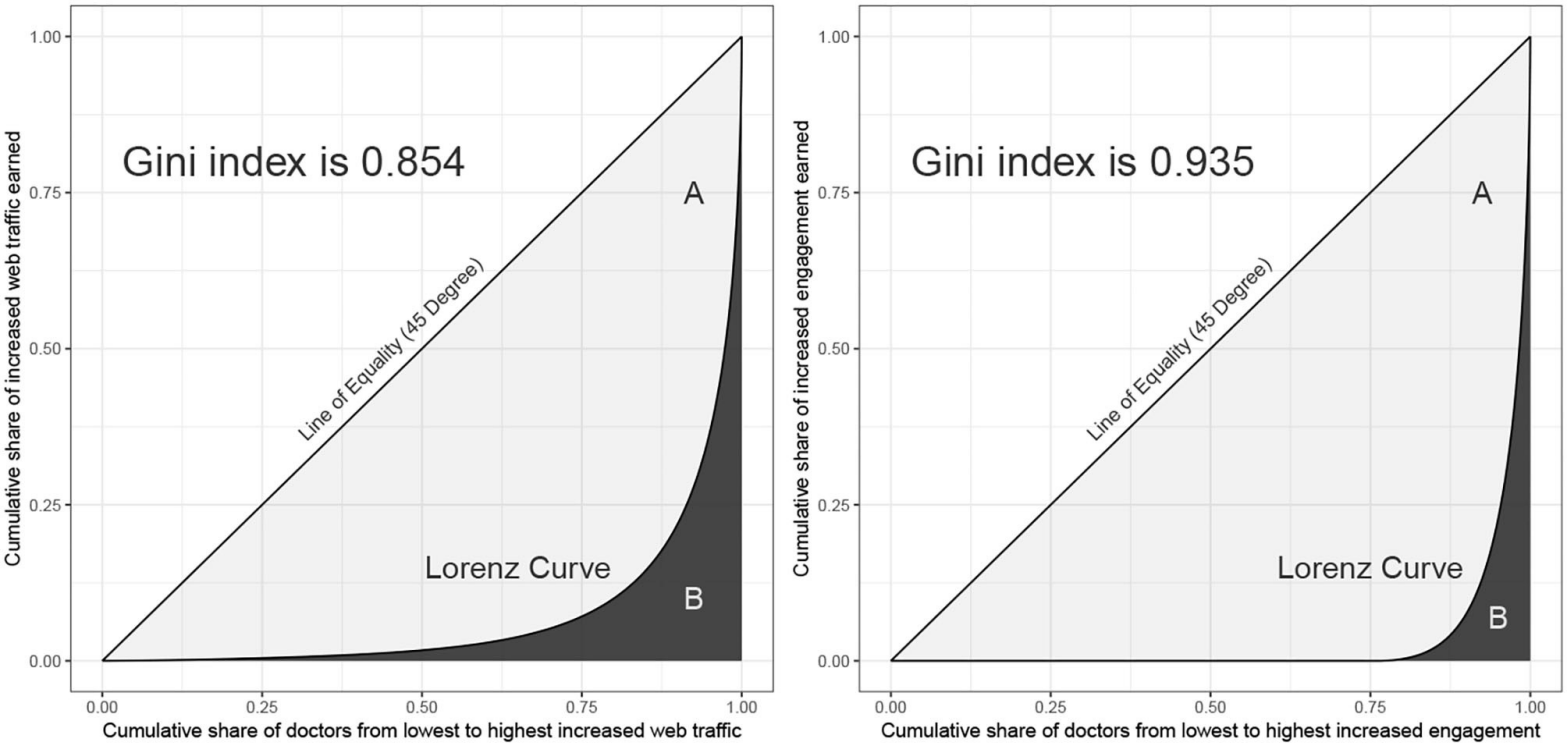
Lorenz curves for increased web traffic and increased engagement.

In addition to noting the above intuitive results, we formally introduce the Lorenz curve and the Gini index to analyze the web traffic and engagement distributions. The Lorenz curve, proposed by the economist Max Lorenz in 1905, is a graphical representation of income inequality. In this income case, the horizontal axis shows the percentile of the population based on income, and the vertical axis shows the corresponding cumulative income. For example, an x-value of 0.9 and a y-value of 0.25 means that the lowest 90% of the population earn only 25% of total income. In our context, Figure 4 shows the Lorenz curve representing the inequalities of increased web traffic and increased engagement. The results verify that most of the increased web traffic (increased engagement) is generated by a small number of doctors with higher levels of increased web traffic (increased engagement). The degree of distributed inequality can be effectively measured by means of the Gini index, which is a ratio of the area between the line of equality and the Lorenz curve (marked *A* in Figure 4) over the area of the triangle under the line of equality (marked *A* and *B* in the Figure 4). Therefore, the Gini index is equal to *A*/(*A*+*B*) and ranges from 0 to 1, with 0 indicating perfect equality and 1 indicating perfect inequality. The Gini indexes for increased web traffic and increased engagement are 0.854 and 0.935, respectively, which indicates extreme inequality. It is thus clear that there are indeed inequalities in the OHC. Next, we determine whether this phenomenon can be explained by the Matthew effect.

### 4.3. Evidence of the Matthew Effect

The Matthew effect in our context represents the idea that doctors who already have advantaged status usually generate more web traffic or engage in more community activities. In particular, we explore whether doctors' activating the web page earlier or having generated more cumulative web traffic (engagement) leads to more increased web traffic (engagement) in the subsequent period. First, we report the effect of tenure on increased web traffic and engagement in Table 3. We divide all doctors equally into 10 groups according to their tenures, from short to long, and denote these as Group 1 to 10. Thus, doctors in Group 1 are the ones who only recently activated their web pages, and their average tenure is 125.5 days, while doctors in Group 10 have had their web pages activated for a considerable period of time, with an average tenure of 3,143.6 days. The influence of tenure on increased web traffic is clear. The average increased web traffic continues to grow from Group 1 to 10. For Group 10, we employed a two-sample *t*-test to examine whether its mean was greater than that of any other group, and results for all comparisons are significant (*p* < 0.001). Nevertheless, as shown in the previous section, a small number of doctors have greatly increased web traffic, which might cause the means to be less meaningful. Therefore, we further calculate the ranking of each doctor, the doctor with the greatest web traffic ranking first. We find the average ranking becomes smaller from Group 2–10, which is almost consistent with the result of the means. Following a similar procedure, we also investigate the influence of tenure on increased engagement and present the results in Table 3. Doctors in Group 10 also have the largest average increased engagment and the smallest average rank, implying that doctors will continue to engage in the OHC to maintain their status. However, it is interesting to further explore the outcomes of Group 1. The doctors in Group 1 have activated their web pages only in the past year, and their average engagement is higher than that of the doctors in Groups 2–5. This indicates that doctors were more willing to contribute to the OHC in the first year, but they gradually became less enthusiastic about their OHC activities over time. In summary, these results support Hypothesis 1a but only partially support Hypothesis 2a.

**Table 3:** The effect of tenure on increased web traffic and engagement.

	**Tenure**	**Increased web traffic**		**Increased engagement**	
**Group**	**Mean**	**Mean**	**Rank^a^**	**Mean**	**Rank^b^**
1	125.4	1542.5	82445.4	74.0	66501.1
2	320.5	1807.5	87252.3	52.8	72474.2
3	440.0	2333.0	84248.0	47.5	73751.4
4	548.6	3614.6	78272.0	63.0	71456.3
5	739.6	4946.7	75111.0	63.7	71481.5
6	1117.9	6294.7	65750.6	95.3	67552.6
7	1683.2	6342.8	63327.4	80.3	69309.5
8	2153.4	8424.1	59100.3	88.1	69331.9
9	2601.9	13568.8	52597.6	99.4	67574.6
10	3143.6	20968.8^c^	46942.0	122.7^c^	65742.4

*All doctors are equally divided into 10 groups according to their tenures, from short to long*.

a *Calculating the ranking for each doctor based on the increased web traffic, this column reports the average ranking for each group*.

b *Calculating the ranking for each doctor based on the increased engagement, this column reports the average ranking for each group*.

c * The mean of Group 10 shows a significantly greater effect than any other group (p < 0.001) through the two sample t-test*.

**Figure 5:**
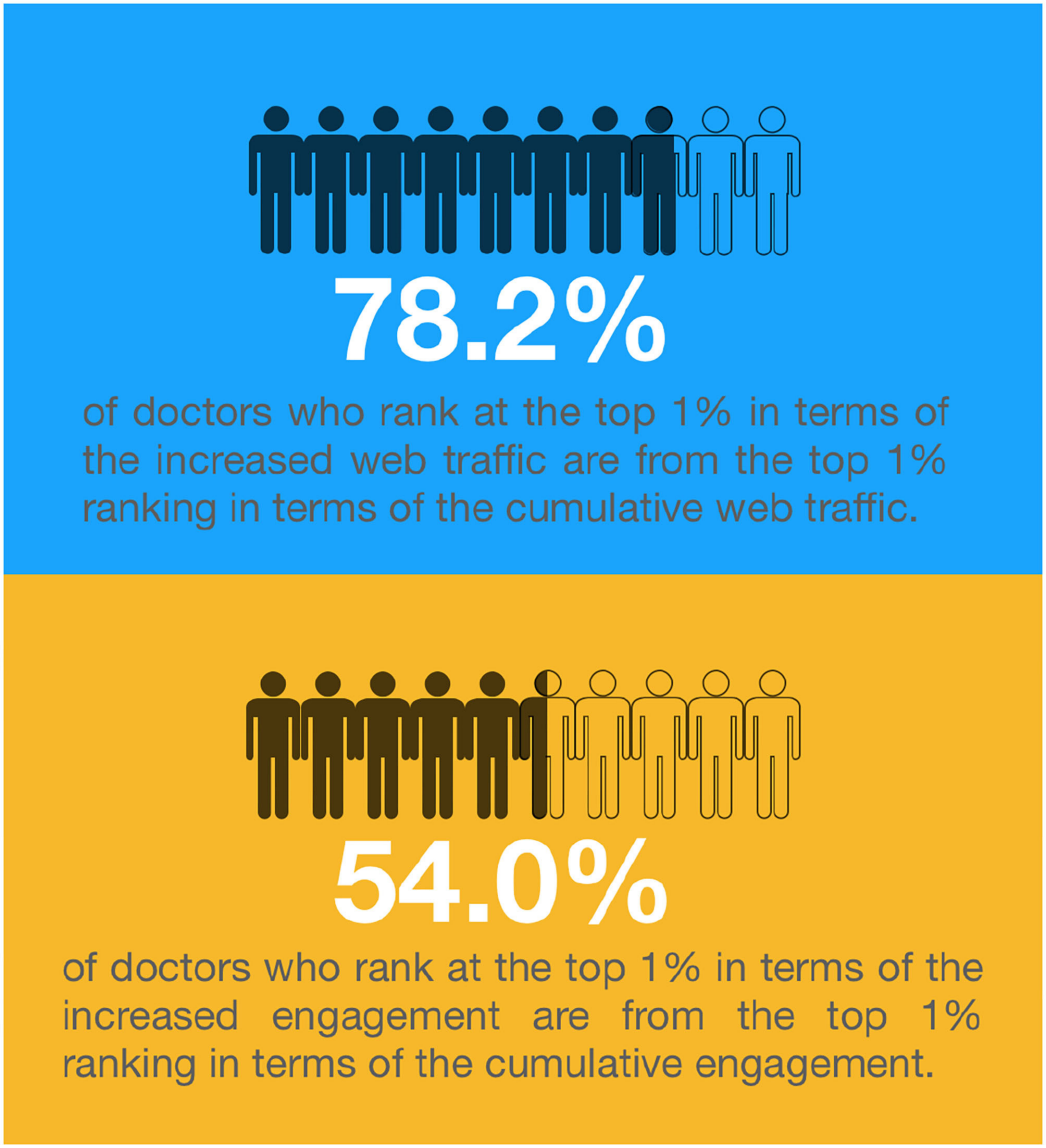
Influences of cumulative web traffic and cumulative engagement.

We then investigate the question of whether doctors with higher cumulative web traffic (engagement) will enjoy more web traffic (engagement) in the subsequent time period. First of all, we provide a short overview of this issue as Figure 5, which reveals that 78.2% (54%) of doctors who rank at the top 1% in terms of increased web traffic (engagement) are from the top 1% in ranking in terms of cumulative web traffic (engagement). In terms of the Matthew effect, these results can be explained by the fact that doctors who initially had the highest 1% advantage continue to increase their advantage in the next phase. Formally, we use the Spearman's rank correlation coefficient to verify the relationship between cumulative values and increased values. For the entire sample, the correlation coefficient is 0.854 (0.599) for cumulative and increased web traffic (engagement). Moreover, even when the doctors are divided into 10 groups as shown in Table 3, this relationship is robust in each group. We report the related results, in which all correlation coefficients are statistically significant, in Table 4. Accordingly, these results support Hypothesis 1b and Hypothesis 2b.

**Table 4:** Results of Spearman's rank correlations.

**Group**	**Corr(*WT*_t-1_, *ΔWT*_t_)**	**Corr(*EG*_t-1_, *ΔEG*_t_)**
1	0.891^***^	0.649^***^
2	0.922^***^	0.602^***^
3	0.940^***^	0.569^***^
4	0.948^***^	0.602^***^
5	0.954^***^	0.601^***^
6	0.957^***^	0.675^***^
7	0.947^***^	0.631^***^
8	0.959^***^	0.597^***^
9	0.966^***^	0.619^***^
10	0.972^***^	0.671^***^
Total	0.854^***^	0.599^***^

^*^p < 0.05; ^**^p < 0.01; ^***^p < 0.001.

### 4.4. Impacts of the Reward Mechanism

In order to explore whether the reward mechanism helps to improve the inequality described above, we group doctors according to their specific expertise on diseases, which is indicated by the voting of patients on the Haodf website. When a doctor has received at least one vote for a specific disease *j*, we assign this doctor into group *j*. In other words, each doctor might belong to zero or more than one disease type groups. If one disease is represented as one group, we have a total of 1,023 groups. However, we deleted groups with fewer than 30 doctors, and we finally retained 823 groups in our sample. Intuitively, a doctor's having a higher proportion of votes in a specific disease usually indicates that a higher proportion of her or his web traffic is related to this disease. Further, this doctor might also spend more energy on some activities related to this disease. Therefore, we can reasonably estimate a doctor's web traffic and engagement in a specific disease by means of the vote weights. For example, if a doctor obtains 100 votes including votes for diseases A (50 votes), B (30 votes), and C (20 votes), her or his vote weights for these diseases will be 0.5, 0.3, and 0.2, respectively. Suppose her or his increased web traffic is 200; then the corresponding adjusted increased web traffic for diseases A, B, and C will be 100, 60, and 40, respectively. For convenience, we use the superscript ^*^ to indicate that a variable has been adjusted by the vote weights; for example, the cumulative web traffic and engagement adjusted by the vote weights at time *t* are denoted as $WT_{t}^{*}$ and $EG_{t}^{*}$, respectively.

Given one individual-level variable in these 823 groups, we use Gini() and Avg() to calculate the corresponding Gini index and average, respectively. For example, Gini($WT_{t}^{*}$) is the Gini index for the distribution of the adjusted cumulative web traffic, and Avg(TENURE_*t*_) is the average length of doctors' tenures in one specific group. In addition, we also define the variable SIZE_*t*_ as the number of doctors belonging to the group at time *t*. In this way, several group-level variables used in the regression analysis are defined in Table 1. Accordingly, we develop two regression models, Model (1) and Model (2), to analyze the effect of the reward mechanism on inequality of increased web traffic and engagement. Specifically, Model (1) and (2) are expressed as


(1) $$\begin{array}{l} \text{Gini}(\Delta WT_{t}^{*})=\beta_{0}+\beta_{1}\text{Gini}(WT_{t-1}^{*})+\beta_{2}{\text{SIZE}}_{t-1}\\ \;+\beta_{3}{\text{Avg(TENURE}}_{t-1})+\beta_{4}{\text{Avg(RATING}}_{t-1})\\ \;+\beta_{5}\text{Gini}(\Delta PR_{t-1})+\beta_{6}\text{Gini}(\Delta MR_{t-1})+\varepsilon_{t} \end{array}$$

and


(2) $$\begin{array}{l} \text{Gini}(\Delta EG_{t}^{*})=\beta_{0}+\beta_{1}\text{Gini}(EG_{t-1}^{*})+\beta_{2}{\text{SIZE}}_{t-1}\\ \;+\beta_{3}{\text{Avg(TENURE}}_{t-1})+\beta_{4}{\text{Avg(RATING}}_{t-1})\\ \;+\beta_{5}\text{Gini}(\Delta PR_{t-1})+\beta_{6}\text{Gini}(\Delta MR_{t-1})+\varepsilon_{t}. \end{array}$$

It is worthwhile to note that both models represent the relationship of the dependent variable at time *t* and several independent variables at time *t*−1. Accordingly, the relevant results can be interpreted as indicating whether the state of the previous independent variable will cause the dependent variable to change in the next period.

We report regression coefficients, standard error, and significance levels for all the variables of Model (1) in Table 5. First, we set Model (1) without the impact of the reward mechanism. As indicated by the corresponding outcomes shown in column (1a), the coefficients of Gini($WT_{t-1}^{*}$) and Avg(TENURE_*t*−1_) are significantly positive, and the coefficient of Avg(RATING_*t*−1_) is significantly negative. Specifically, these empirical results indicate that if the doctors in a group have a higher inequality of cumulative web traffic, a higher average tenure with the OHC, or a lower average rating, their inequality of increased web traffic in the subsequent period will be more severe. When Gini(Δ*PR*_*t*−1_) and Gini(Δ*MR*_*t*−1_) are added into our model, the adjusted *R*^2^ rises from 0.659 to 0.747, which means that these two variables can effectively explain more of the variation of the dependent variable. The relevant results are displayed in column (1b). Besides the fact that the coefficient of SIZE_*t*−1_ becomes significant, the effects of the other variables are quite robust. A negative coefficient of SIZE_*t*−1_ implies that more doctors in a group might ameliorate the inequality of increased web traffic. More importantly, since the coefficients of both Gini(Δ*PR*_*t*−1_) and Gini(Δ*MR*_*t*−1_) are significantly positive, Hypothesis 3a is supported.

**Table 5:** The impact factors on the inequity of increased web traffic.

	**Dependent variable:**	
	**Gini(** $\Delta WT_{\text{t}}^{*}$ **)**	
	**(1a)**	**(1b)**
Gini($WT_{t-1}^{*}$)	0.7962^***^	0.6946^***^
	(0.0232)	(0.0211)
SIZE_*t*−1_	−0.000005	−0.00001^***^
	(0.000004)	(0.000003)
SIZE${}_{t-1}^{2}$		
Avg(TENURE_*t*−1_)	0.00005^***^	0.0001^***^
	(0.000005)	(0.000004)
Avg(RATING_*t*−1_)	−0.1073^***^	−0.0354^**^
	(0.0133)	(0.0123)
Gini(Δ*PR*_*t*−1_)		0.1112^***^
		(0.0287)
Gini(Δ*MR*_*t*−1_)		0.2762^***^
		(0.0270)
Constant	0.4511^***^	−0.0701
	(0.0564)	(0.0585)
Observations	823	823
R^2^	0.6605	0.7487
Adjusted R^2^	0.6589	0.7469

^*^p < 0.05; ^**^p < 0.01; ^***^p < 0.001.

Similar results for Model (2) are shown in Table 6. The coefficient of Gini($WT_{t-1}^{*}$) is significantly positive, and the coefficient of Avg(RATING_*t*−1_) is significantly negative, whether Gini(Δ*MR*_*t*−1_) and Gini(Δ*MR*_*t*−1_) are considered or not. However, because doctors with particularly small or large tenures also have higher increased engagement, as indicated in Table 3, the impact of Avg(TENURE _*t*−1_) in Model (2) is unstable. When Gini(Δ*PR*_*t*−1_) and Gini(Δ*MR*_*t*−1_) are included, the adjusted *R*^2^ rises from 0.606 to 0.808. That means as a result of the addition of the reward mechanism variables in Model (2), that model has improvements in comparison to Model (1) in terms of the adjusted *R*^2^. The coefficients of Gini(Δ*PR*_*t*−1_) and Gini(Δ*MR*_*t*−1_) are not only significantly positive but also clearly larger than the corresponding coefficients in Model (1), implying that the reward mechanism might play a more important role in ameliorating the inequality of increased engagement. Based on the above evidence, Hypothesis 3b is also supported.

**Table 6:** The impact factors on the inequity of increased engagement.

	**Dependent variable:**	
	**Gini(** $\Delta EG_{\text{t}}^{*}$ **)**	
	**(2a)**	**(2b)**
Gini($EG_{t-1}^{*}$)	0.6201^***^	0.2213^***^
	(0.0240)	(0.0218)
SIZE_*t*−1_	0.000001	−0.00001^***^
	(0.000005)	(0.000003)
Avg(TENURE_*t*−1_)	−0.00002^***^	0.00002^***^
	(0.00001)	(0.000005)
Avg(RATING_*t*−1_)	−0.1394^***^	−0.0521^***^
	(0.0165)	(0.0120)
Gini(Δ*PR*_*t*−1_)		0.2390^***^
		(0.0287)
Gini(Δ*MR*_*t*−1_)		0.5688^***^
		(0.0278)
Constant	0.9116^***^	0.1333^*^
	(0.0695)	(0.0566)
Observations	823	823
R^2^	0.6080	0.8092
Adjusted R^2^	0.6061	0.8078

^*^p < 0.05; ^**^p < 0.01; ^***^p < 0.001.

## 5. Conclusions

### 5.1. Empirical Findings and Managerial Implications

Online healthcare communities can reduce medical information asymmetry by means such as helping patients find the right doctor or obtain medical knowledge, as well as by allowing patients to communicate directly with doctors online. Therefore, they are a potential solution to rural-urban healthcare differences (73), especially in developing countries such as China. To promote the steady development of OHCs, several studies have been carried out from the perspective of patients or doctors (9, 10, 36, 37). However, this study is the first from the points of view of OHC managers to observe and analyze the status of OHC development. There are three innovations or contributions made by this study. First, based on a large sample extracted from 139,037 doctors' web pages, the Gini indexes of increased web traffic and increased engagement are as high as 0.854 and 0.935, respectively, which means that inequality in the OHC is very serious. In other words, only a small proportion of doctors can attract patients to browse content in the OHC or are willing to actively participate in the OHC's activities, and OHC managers should realize that this phenomenon is certainly not conducive to the development of the OHC.

Second, we discover that inequality in OHCs can be explained by the Matthew effect. For web traffic, doctors who activated their web pages earlier or had larger cumulative web traffic possess a distinct advantage and are more likely to have more web traffic in the next phase. This brings a dilemma for managers because these key few doctors attract the vast majority of users, but relying on them will reduce the diversity of the OHC and increase operation risk. It is well known that one should never put all one's eggs in one basket. Nevertheless, even if the OHC managers are already aware of this problem, it is difficult to change the habits of a large number of patients. In addition, doctors with more patients are more likely to attract a new patient's attention through the recommendations of old or existing patients. Therefore, the OHC managers have difficulty finding an effective way to overcome the advantage of doctors in web traffic. A similar phenomenon also occurs in the increased engagement of doctors, but the influence of a doctor's tenure on increased engagement is only partially supported by our empirical results, as shown in Table 3. Specifically, when a doctor's tenure is longer, the increased engagement is higher, but when a doctor's tenure is particularly short, the increased engagement is still relatively high. The former effect comes from the doctor's effort to maintain her or his status in the community (56), while the latter is related to the doctor's initial enthusiasm (59, 60). Based on these outcomes, we suggest that OHC managers should develop special mechanisms to allow new doctors to maintain their enthusiasm and to motivate them continue to participate in OHC activities. As doctors experience more and more engagement, the likelihood that they will continue to engage in the OHC increases.

Finally, regression analysis is used to examine the effects of the reward mechanism on inequality in the OHCs. From the results shown in Tables 5, 6, we can conclude that the inequality of psychological or material rewards causes the inequality of OHCs to become worse. Accordingly, managers must consider how to properly distribute rewards and avoid their excessive concentration on a small number of doctors. In fact, because the marginal utility of doctors' receiving rewards is diminishing (10), appropriately reducing the rewards of certain doctors, who have already received sufficient rewards, would not have much impact on them. In practice, the factors related to assigning rewards could be included in the doctor recommendation algorithm, so as to make it easier for patients to obtain information about doctors who have received fewer rewards. Although the Matthew effect cannot be completely eliminated in OHCs, developing an optimal reward mechanism should mitigate it rather than enhance it.

### 5.2. Limitations and Directions for Future Research

In this section, we indicate the limitations of this study and suggest potential directions for future research. First, the advantage of this study is the use of a large sample collected from the most popular OHC in China. Nevertheless, compared to traditional questionnaires, this kind of data collection approach results in the acquisition of variables that are limited to the design of the existing website. Moreover, as with any other Internet platform, it is difficult for the Haodf website to avoid shilling attacks (74) even though it has adopted multiple methods to prevent these attacks such as strictly checking the authenticity of the doctor. Therefore, ways to combine external sources with the original sample, such as questionnaires, and ways to reduce the impact of fraudulent data should be noted in future studies. Second, although the Haodf website is the most popular OHC in China, it is undeniable that there are many other famous OHCs. Due to the cost of data acquisition, we did not conduct a comparison of different platforms. If the relevant data are available, future studies can explore how the website designs affect inequality in various OHCs.

Furthermore, due to the lack of sufficient data period, this study does not discuss the long-term relationship between the doctor's web traffic and engagement. Such a relationship might be meaningful in investigating the different situations of doctors. For example, some doctors have less increased web traffic and more increased engagement, which means they do a lot for the OHC, but only a few patients are interested in them. In the opposite situation, some doctors have not made many contributions, but they can attract a large number of patients. In the long run, a doctor may currently be in the former but after some time she or he will be in the latter situation. To understand how the situation of the doctor changes and what factors induce these changes would also be helpful for the development of OHCs.

Finally, most doctors in OHC only work part-time, offline features of the Chinese health-care system influence OHC, such as patient preference for elite hospitals, hierarchical diagnosis and treatment policies, or unequal distribution of medical resources. However, due to the limitations of our collection of offline data sources, this study does not provide more information about the offline health care in China. If offline data are available, future studies can explore how website designs affect inequality in various OHCs. By doing so, the findings of interaction between offline and online health care would provide insightful implications for OHC policy makers in China.

## Data Availability Statement

The original contributions presented in the study are included in the article, further inquiries can be directed to the corresponding author.

## Author Contributions

Y-TH: investigation, resources, methodology, and writing–original draft preparation. Y-LC: writing–reviewing and editing and conceptualization. J-NW: visualization, formal analysis, investigation, and conceptualization. RD: data curation and writing–original draft preparation. All authors contributed to the article and approved the submitted version.

## Funding

This work was supported by Ministry of Education Project of Humanities and Social Sciences (No. 20YJCZH199).

## Conflict of Interest

The authors declare that the research was conducted in the absence of any commercial or financial relationships that could be construed as a potential conflict of interest.

## Publisher's Note

All claims expressed in this article are solely those of the authors and do not necessarily represent those of their affiliated organizations, or those of the publisher, the editors and the reviewers. Any product that may be evaluated in this article, or claim that may be made by its manufacturer, is not guaranteed or endorsed by the publisher.

## Footnotes

^1^In 1906, the Pareto principle was first proposed by the Italian economist, Varefredo Pareto. He observed that approximately 80% of the land in Italy was owned by 20% of the population. From the statistical views, many empirical distributions of the effects in business exhibit this power-law behavior (28).

^2^Due to changes in website design, contribution scores have not been made public on the Haodf website since October 2018.
